# Definition of an Enhanced Map-Matching Algorithm for Urban Environments with Poor GNSS Signal Quality

**DOI:** 10.3390/s16020193

**Published:** 2016-02-04

**Authors:** Felipe Jiménez, Sergio Monzón, Jose Eugenio Naranjo

**Affiliations:** University Institute for Automobile Research (INSIA), Ctra. Valencia, Km. 7, Madrid 28031, Spain; felipe.jimenez@upm.es (F.J.), sergio.monzon3@gmail.com (S.M.)

**Keywords:** satellite positioning systems, Global Navigation Satellite System (GNSS), navigation, information fusion, map matching.

## Abstract

Vehicle positioning is a key factor for numerous information and assistance applications that are included in vehicles and for which satellite positioning is mainly used. However, this positioning process can result in errors and lead to measurement uncertainties. These errors come mainly from two sources: errors and simplifications of digital maps and errors in locating the vehicle. From that inaccurate data, the task of assigning the vehicle’s location to a link on the digital map at every instant is carried out by map-matching algorithms. These algorithms have been developed to fulfil that need and attempt to amend these errors to offer the user a suitable positioning. In this research; an algorithm is developed that attempts to solve the errors in positioning when the Global Navigation Satellite System (GNSS) signal reception is frequently lost. The algorithm has been tested with satisfactory results in a complex urban environment of narrow streets and tall buildings where errors and signal reception losses of the GPS receiver are frequent.

## 1. Introduction

Vehicle positioning is a key element in the performance of numerous driver assistance and safety systems [1]. Depending on the particular demands, higher or lower levels of accuracy are required when positioning the vehicle [2]. Thus, informative applications for navigation systems need not be especially accurate while other systems such as those focused on the improvement of traffic safety might need submetric precisions [3]. Generally speaking, the location of a vehicle is mainly accomplished by satellite positioning systems (e.g., GNSS). With the current satellite positioning technology that can be found in all vehicle positioning systems, that submetric precision is not usually available and there are many situations where the environment causes the GNSS signal to be frequently lost or damaged (e.g., driving among tall buildings or in areas with dense tree cover). Even in less demanding applications, such as navigation systems, there are situations where positioning errors are made when locating the vehicle into a link. These errors come mainly from two sources: errors and simplifications of digital maps and errors in locating the vehicle. From that inaccurate data, the task of assigning the location of the vehicle to a link on the digital map at every instant is carried out by map-matching algorithms whose performance depends on many factors. As of today, locating a vehicle in an urban environment with high street density cannot be achieved at every instant, due to GNSS signal reception losses. 

However, there are several solutions for improving the accuracy of satellite positioning. Differential correction techniques could improve GPS accuracy from 10 to 15 m of error to 1 cm of error. If this differential correction is obtained using only GPS pseudo-range code, it is named Differential GPS (DGPS) and can achieve accuracies of around 1 to 5 m. On the contrary, if it is obtained using the GPS carrier phase information, it is named Real Time Kinematics (RTK). DGPS and its accuracy is around 1 to 10 cm. However, these solutions are not limitless. In [3], the authors pointed out that the previous solutions accumulate a high amount of time without GPS coverage and the high precision levels offered by type 4 or type 5 positioning are available less than 37% of the time in some situations. On the other hand, the solution of using our own base stations for the differential correction has a very limited range and it is only acceptable for obtaining high levels of accuracy near the base station, so it is not an acceptable solution for real traffic conditions. Moreover, the equipment cost must be taken into account, which in addition to the connections required to obtain the differential corrections, makes them an invalid solution for their global implementation at present. In all previous situations the signal reception losses are still occurring and so it is necessary to turn to methods that enable positioning even in those areas. To do so, inertial systems are usually employed, though these systems have a cumulative error [3] that makes their reliability decrease as the travelling distance increases [2]. In particular, [4] analyse the effect of using low-performance and low-cost sensors. In [5], the authors also show that the GPS coverage in central Hong Kong can be less than 20%, which makes it essential to use other systems to keep the user informed of their route.

In dense urban areas it is still difficult to obtain good positioning using a single technology. This problem has led to the introduction of combining multiple positioning techniques. Intelligent Urban Positioning (IUP) is based on combining positioning algorithms augmented with three dimensional mapping techniques for distinguishing between non-line-of-sight (NLOS) and line-of-sight (LOS) signals and multi-constellation GNSS, using signals from all visible satellites. This can be used to predict the blockage and reflection of signals [6]. For example, a method using a 3D city model to improve cross-street GNSS positioning accuracy is presented in [7].

Additionally, digital maps have improved their accuracy, mainly when their construction methods have changed from air pictures [8,9] to instrumented vehicles [10,11]. The use of instrumented vehicles usually includes satellite positioning systems and inertial systems and both measurements are combined [2]. Although both sources are combined, their own particular errors mean the final map is not perfectly accurate. Furthermore, it must be taken into account that optimized storage of spatial road network data involves simplifications that can be larger or smaller according to the kind of approximation employed [11]. 

Finally, as stated in [12], “the process of continually estimating a user’s position on a road segment is known as map matching”. In this process, it has to be taken into account that both information sources (GNSS positioning and digital map data) have associated errors, and therefore the algorithm attempts to found the location of the vehicle on the link that most approaches its real position. 

Efficient map-matching algorithms have many applications apart from the ones focused on the driver assistance systems that have been cited above. For example, for travel behaviour, it is required to unambiguously identify the correct road links followed by the traveller and all these identified links should form a meaningful travel route [13,14]. Depending on the final application, the requirements imposed on the map-matching algorithm could be diverse. In this sense, safety applications require high standards of accuracy and reliability that are not completely essential for personal navigation [15]. The previous systems require real time processing but traveller displacement studies allow offline processing. 

Several map-matching algorithms have been developed up to the present [12,16]. The geometric algorithms are the simpler ones but they do not perform well at junctions, roundabouts and parallel roads [17,18,19,20]. There are three different approaches to geometric algorithms, *i.e.*, point-to-point matching, point-to-curve matching and curve-to-curve matching. However, their simplicity means they do not usually perform well in complex situations. More complex methods are used in topological algorithms, which use the relationship between entities of the digital map such as adjacency, connectivity and containment [21,22,23,24] to select the correct link. Hence, they take into account the previous information of the route to locate the vehicle into a link and allow the algorithm to keep the continuity of the trajectory. Probabilistic algorithms generate a confidence region around a position fix obtained from a GPS receiver, and are used mostly in the decisions concerning junctions [25,26]. Finally, advanced algorithms use Kalman Filters (KF), Extended Kalman Filters (EKF), Fuzzy Logic models (FL), Dempster-Shafer’s mathematical theory of evidence, particle filters or Bayesian inference and outperform the other algorithms in terms of correct link identification and accuracy. Nevertheless, they require more input data and could be relatively slow and difficult to implement in real-time applications [27,28,29,30].

Due to the correlation between the different kinds of algorithms enumerated above, [29] proposed a distinction between simple method algorithms, weighted algorithms and advanced algorithms. The former include geometric and topological algorithms, such as probabilistic algorithms, as long as they do not use historic data of the route. The latter try to improve the map-matching results by applying weights when choosing the correct link in which the vehicle is located. These algorithms reach percentages of correct link identification of between 85% and 98.5%. Nonetheless, their performance is not enough to support some ITS applications. These kinds of algorithms are the ones developed by [21,23,31,32].

Though there are others, some algorithms that offer good performances are mentioned in the following lines. According to [12], the algorithm developed in [30] is one of the most accurate according to the correct number of segment identifications and the accuracy of the location of the vehicle in each link. This algorithm, which uses an integrated GPS/DR sensor identifies 98%–99% of links correctly. Another algorithm, developed in [31] reaches a percentage of around 96% of correct link identification. The complex algorithm developed in [32] ranges from 97% to 99.8%. This algorithm uses inputs generated by an integrated GNSS/DR and height data obtained from a Digital Elevation Model (DEM). 

The difference between real-time algorithms and post-processing algorithms should be pointed out. The former, such as those focused on in-vehicle navigation systems should be simple and not computationally intense. The latter use all the position fixes generated by a GNSS along a route as an input, and compare them with the digital map data to produce the whole route travelled as an output. In this last scenario, rapid computer processing is not essential, so the algorithms can be more complex and, therefore, the results could outperform those executed in real-time. 

Finally, due to the errors in GNSS position and digital maps, there can be a high degree of uncertainty associated with the map-matched locations and a quality indicator representing the level of confidence in map-matched locations could be necessary. An empirical method to derive the integrity of a map-matched location is proposed in [33].

In this research, a real-time map-matching algorithm is put forward. It is focused on environments that influence negatively on the GNSS signal reception (reception losses or accuracy degradation) and requires little information from the digital map. It can also work without using additional information from inertial systems, which allows it to be implemented in after-market navigators. The algorithm is tested in an urban environment where the GNSS coverage is poor and there are frequent signal reception losses. Likewise, the use of the speed information as a new input is tested, even without needing high accuracy in its measurement.

## 2. Map Matching Algorithm Proposal

### 2.1. Specifications 

The map-matching algorithm developed in this research tries to simultaneously improve some of the limitations of other algorithms. Specifically, the following characteristics have been defined for it:

- The algorithm should be implemented in real time and with limited computational means.
- The algorithm is based on satellite positioning and digital map data, without using inertial sensors because these will limit its implementation in after-market devices.
- Optionally, it will include the possibility to use speed information, if it is available, to know when the vehicle is moving and when not. Using inertial sensors for speed and yaw angle could improve the final results but could increase the hardware cost if high performance sensors are integrated [4]. Furthermore, the aim is to develop an algorithm that could be implemented in any navigation device, and not all of them contain these sensors, even low performance ones.
- The information contained in the digital map has to be as simple as possible. The necessary information includes *x*-*y* coordinates, the legal turns and the segments priority.
- After making a change of segment, the algorithm should check for some time if it has been a good decision or if the vehicle needs to be relocated to another link.
- The algorithm should recover the positioning of the vehicle quickly after long periods of time without GPS coverage.
- In the event of an error in the positioning of the vehicle in a link, the algorithm should be able to detect the erroneous decision and correct it in a few seconds taking into account the previous correct location.
- The algorithm should be capable of working even with degraded GNSS signals.

### 2.2. Description

The designed algorithm uses topological information from the digital map, as well as historical information of the route. Basically, the map is divided into segments (defined between crossings), formed by links or sub-segments (for all two consecutive points on the map a link is created). The algorithm could be divided into four main steps:

1. Candidate segments and links definition
2. Preliminary segment selection:Topological and historical links parameters and final weight calculation of the segments
3. Definitive segment selection: (1) Correction of the belonging parameter impact; (2) Correction of inappropriate segment change
4. Vehicle positioning on the digital map

#### 2.2.1. Step 1: Candidate Segments and Links Definition

For each GNSS positioning, the segments included in a confidence region (circumference of radius R) are identified as candidates. Each link contained in a candidate segment within the confidence region becomes a candidate link of that segment. 

#### 2.2.2. Step 2: Preliminary Segment Selection

In order to select the segment in which the vehicle is located, some parameters are calculated according to the physical characteristics and the relationship with the GNSS-generated point. Taking into account previous approaches found in the literature review of geometric, topological and probabilistic algorithms [17,18,19,20,21,22,23,24,25,26] and tests that provide the relative relevance of each parameter on the final positioning results, the following have been chosen:

1. Distance parameters:
  - Point-to-point average distance parameter: It uses the average distance between the two extreme points of the candidate link and the location given by the GNSS receiver. This value is useful to improve situations where the perpendicular distance is small but the link is not actually near the GNSS location.
  - Perpendicular distance parameter: It uses the perpendicular distance between the GNSS positioning and the line that contains the candidate link.
2. Parameters depending on the movement history:
  - Direction parameter: It compares the difference between the GNSS trajectory and the link trajectory by calculating the angle between them. To estimate the trajectory of the vehicle the last GPS positions are taken into account, instead of just using the last two. Should the GNSS reception losses be longer than 1 s, this parameter decreases its relevance linearly because its relevance is lower as the time without the GNSS signal increases.
  - Segment belonging parameter: It assigns values to the candidate links according to their priority in a hypothetical junction (stored in the digital map information) and penalizes those links that are not physically accessible or those that involve illegal turns. At a junction, those links that involve going straight (keeping in the same street that is, not doing any turns) are considered priority links and get a higher value than those legal turns not considered priority links. The illegal turns receive a negative value to penalize them and avoid their being taken as the vehicle location by the algorithm. However, if the user makes an illegal turn, the algorithm will locate the vehicle correctly after a few seconds. Finally, the links that belong to the segment where the vehicle was positioned before receive the highest value. Like the Direction parameter, should the GNSS reception losses be longer than 1 s, this parameter decreases its relevance linearly because its relevance is lower as the time without the GNSS signal increases.
3. Additional parameter for segments:
  - Perpendicular parameter: An additional value is assigned to a candidate segment in case the perpendicular projection of the GNSS point on one of the lines defined by each candidate link is among the two points of the digital map that define the link.

The sum of the first four parameters referred to links gives the final weight for each candidate link, and the average value of all links contained in a candidate segment, adding the perpendicular parameter, gives the final segment weight. Table 1 shows a summary of all the parameters and the final equation for the final segment weight. Table 2 includes the nomenclature used in the equations. The algorithm obtains a preliminary decision in which the vehicle is located in the segment with the highest weight.

**Table 1:** Parameters included in the map-matching algorithm.

Parameters		
Point-to-point distance	$W_{d2}=K_{d}\cdot \left[1-\frac{\bar{d}\left(GNSS,link\;k\right)}{R}\right]$	(1)
Perpendicular distance	$W_{d}=K_{d2}\cdot \left[1-\frac{d\left(GNSS,link\;k\right)}{R}\right]$	(2)
Direction	$W_{a}=K_{a}\cdot \left|cos\;\propto_{k}\right|$	(3)
Belonging	$W_{b}=K_{b}\cdot \beta_{k}$	(4)
Perpendicularity	$W_{p}=\{\begin{array}{c} K_{p}\;if\;the\;condition\;is\;satisfied\\ 0\;if\;the\;condition\;is\;not\;satisfied \end{array}$	(5)
**Segment *s* Final Weight**		
$W_{T,s}=\frac{\sum_{k=1}^{n_{s}}\left(W_{a,k}+W_{b,k}+W_{d,k}+W_{d2,k}\right)}{n_{s}}+W_{p,k}$		(6)

**Table 2:** Nomenclature.

d(GNSS, link k)	Distance between the GNSS point and link *k*
$\propto_{k}$	Angle between the GNSS trajectory and the link *k* trajectory
$\beta_{k}$	Denotes link *k* priority
$n_{s}$	Number of candidate links contained in segment *s*
$K_{d},\;K_{d2,}K_{a},\;K_{b},\;K_{p}$	Parameter coefficients Ka, Kb={NV if tl≤1s(NV/9)(tl−10) if 1s≤tl≤10s 0 if tl≥10s NV≡nominal value $t_{l}\equiv GNSS\;signal\;reception\;loss\;time$

#### 2.2.3. Step 3: Definitive Segment Selection:

In order to corroborate the current segment selection, some scenarios of possible previous incorrect segment selections are considered and corrections can be performed in a short period of time.

The fact that the belonging parameter has a high influence when calculating the average value of the segment can cause the algorithm to take a little more time than necessary to detect when the vehicle has turned at a junction, so the segment where the vehicle is located has changed. To solve this problem, the algorithm also calculates the weights of all candidate segments without considering the belonging parameter:

(7) $${W\prime }_{T,s}=\frac{\sum_{k=1}^{n_{s}}\left(W_{a,k}+W_{d,k}+W_{d2,k}\right)}{n_{s}}+W_{p,k}$$

where *W'_T,s_* is the weight of the segment *s* without considering the belonging parameter.

This way, if this new total weight is higher for a different segment *s_’_* than for the one that has been chosen to locate the vehicle, the algorithm starts to store an uncertainty variable that adds to the first calculated weight of the segment (which considers the belonging parameter) to check if now the first weight plus the uncertainty variable are higher than the original weight assigned to the chosen segment. Then, the algorithm applies the following decisions:

If:

(8) $${W\prime }_{T,s\prime }>{W\prime }_{T,s}$$

if:

(9) $$W_{T,s\prime }+U_{s\prime }>W_{T,s}\;\Rightarrow \;Change\;of\;segment\;from\;s\;to\;s\prime$$

where *U_s’_* is the uncertainty variable of segment *s’*.

else:

(10) $$U_{s\prime }=U_{s\prime }+\Delta U_{s\prime }\;and\;there\;is\;no\;change\;of\;segment$$

else there is no change of segment and the vehicle is located in segment *s*.

Thanks to the uncertainty, the change of segment will be done quickly. Moreover, to let the program restore any mismatching in a quick way, in the event it has made an incorrect change of segment, when a change of segment is made, and if Equation (8) is satisfied, the algorithm calculates, for each point generated by the GPS, the total weights of the candidate segments not only considering the new belonging parameter but also the belonging parameter that the candidate segments had before the change was made. Therefore, considering the following criteria, the algorithm is capable of restoring from its positioning errors quickly. If Equation (8) is satisfied the algorithm applies the following calculations and decisions:

(11) $${W\prime \prime }_{T,s}=\frac{\sum_{k=1}^{n_{links}}\left(W_{a,k}+W_{b\prime ,k}+W_{d,k}+W_{d2,k}\right)}{n_{links}}+W_{p,k}$$

where *W''_T,s_* is the weight of the segment s considering the belonging parameter that this segment had before the change of segment was made (symbolized by *W_b',k_*):

(12) $$if\;{W\prime \prime }_{T,s\prime }>{W\prime \prime }_{T,s}\;\Rightarrow \;Change\;of\;segment\;from\;s\;to\;s\prime$$

else there is no change of segment and the vehicle is located in segment ***s***.

**Figure 1:**
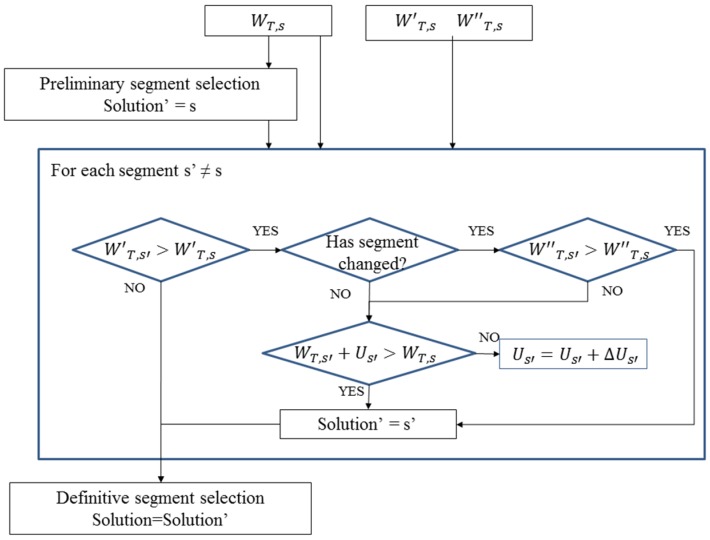
Preliminary segment verification algorithm.

Figure 1 shows a diagram that illustrates the decision algorithm that corroborates or changes the preliminary selection of segment *s*.

#### 2.2.4. Step 4: Vehicle Positioning on the Digital Map

Finally, once the preferred segment is selected, the positioning of the vehicle is carried out with the following criteria:

- If any of the candidate links of the chosen segment contain the perpendicular projection of the GNSS positioning between their two extremes, then this point is used to locate the vehicle.
- If the condition above does not apply for any link, the nearest point of the digital map to the GNSS-generated point that belongs to the selected segment is assigned to locate the vehicle.

## 3. Practical Application

### 3.1. Urban Scenario and Digital Map

**Figure 2:**
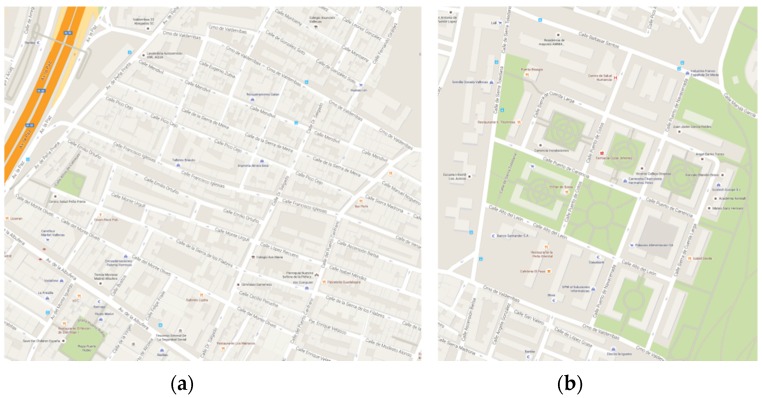
Test areas. (**a**) Route 1; (**b**) Route 2.

To test the algorithm’s performance, two areas of central Madrid (Spain) were selected where the GNSS coverage is poor, with frequent signal reception losses and signal damage because of the constant change of satellites detected caused by the narrow streets with tall buildings and dense tree cover (Figure 2).

The digital map was generated using an instrumented vehicle with the following equipment:

1. Inertial measurement system: (1) Correvit L-CE- non-contact speed sensor to measure speed and the distance travelled; (2) RMS FES 33 gyroscopic platform to provide measurement of the angles drawn about three axes.
2. Astech G12 GPS autonomous receiver with an update frequency of 10 Hz.

The GPS position fixes of the analysed zones have high uncertainties. Specifically, on route 1, the GPS coverage was active 46.8% of the time, and on route 2 it reached a 71.1% value. Nevertheless, if the wrong positioning received by the GPS receiver, that is, those positions that are too far from the actual location to be considered valid, are eliminated, the percentages of relatively good GPS position fixes decrease to 33.5% and 56.0%, respectively. In both cases, they are low values consistent with other studies [5]. For this reason, the digital map was generated as stated in [2,11]. Nonetheless, in order to reduce the cumulative error of this process, and because the results obtained by the inertial sensor need to be turned and translated [2], this turn is done by steps to minimise the deviations between the generated trajectory and the positioning points obtained by the satellite positioning navigator.

**Figure 3:**
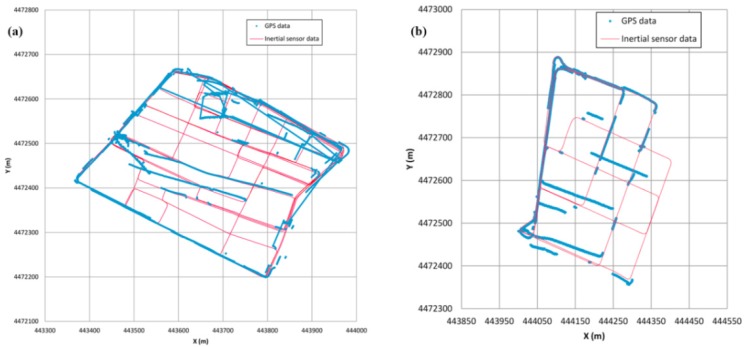
Data adjustment using inertial measurement system and GPS receiver. (**a**) Route 1; (**b**) Route 2.

In Figure 3, it can be seen that, for the adjustment that has been made, when the route passes more than once along a certain street, the different inertial sensor coordinates overlap one another quite exactly, and there are no deviations such as those where the different passes show different orientations, as usually occurs because of the cumulative error of the inertial systems [4].

**Figure 4:**
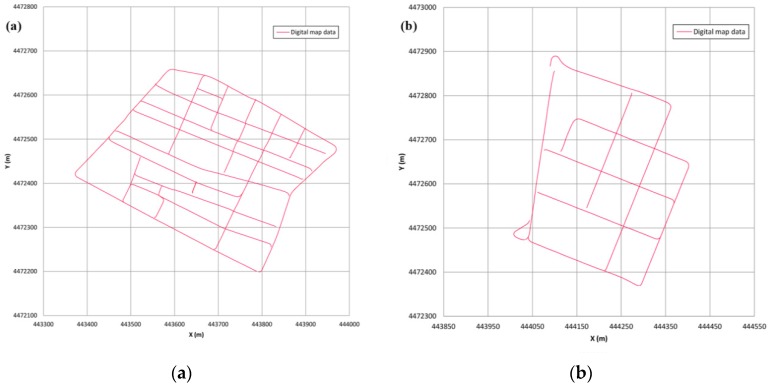
Digital maps for the test areas. (**a**) Route 1; (**b**) Route 2.

Finally, Figure 4 shows the points of the digital map stored with which the map-matching algorithm will be evaluated. In order to test the robustness of the algorithm, intersections in the digital map include some missing data so that poor information in these critical sections is consciously provided. On the other street stretches, points of the digital map are separated by 2 m. 

### 3.2. Results of the Map Matching Algorithm

**Figure 5:**
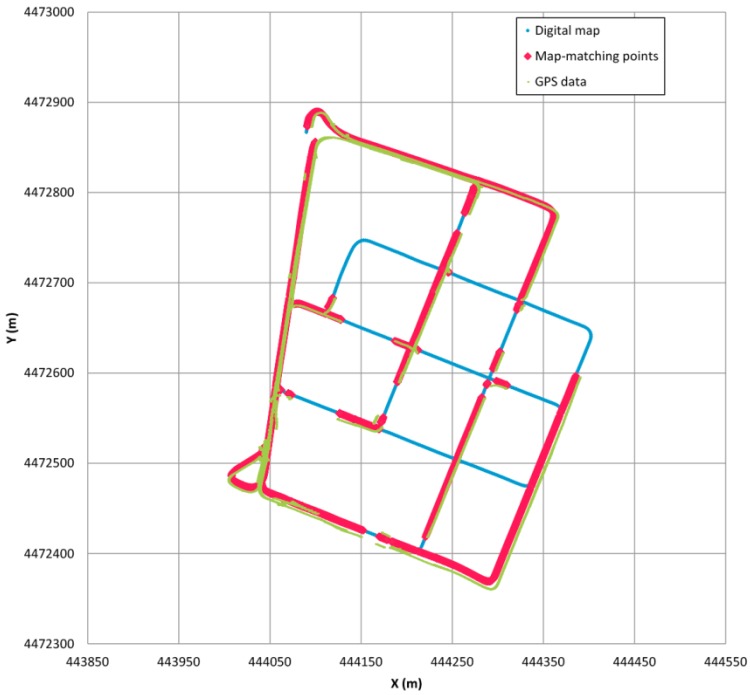
Example of the map-matching algorithm performance.

In order to analyse the performance of the developed algorithm, some tests were carried out in the previously mentioned areas, going along a different route from the one used to create the digital map, to avoid any correlation between them. Besides, the tests were carried out at different times and on different dates from those used to generate the map. The equipment used in the test was the same Astech G12 GPS receiver and additionally the speed of the vehicle was obtained from the vehicle internal communication bus, because its measurement did not need to be too accurate as it was only used to distinguish when the vehicle was moving and when not. The confidence region for this GPS receiver was fixed in a circumference whose radius is 12 m. This value was obtained after completing tests with the receiver in different static locations over a long period of time and analysing the dispersion of the positioning points. The static locations included obstacle-free areas and areas with some high obstacles in the nearby and densely covered areas. The defined circumference comprises more that 75% valid points. Figure 5 shows the results of one of the examples during a test of the position points obtained from the GPS receiver, along with the digital map points and the positioning of the vehicle on it using the map-matching algorithm.

Link identification is considered to be correct when the algorithm assigns a certain GPS positioning to its correct segment on the map. With this consideration it has been observed that the algorithm correct link identification percentage ranges from 98.1% to 99.3%, the higher value being achieved when the speed information is taken as an input. These results are quite satisfactory and are among the ones obtained by the map-matching algorithms developed in [30,32], among others. Unlike these other algorithms, no information is required from inertial sensors (the speed is only used to detect when the vehicle is not moving) and it is quick enough to be executed in real-time, and also, it is possible to have high control over its performance by changing the parameters and weights defined along the algorithm. Additionally, the causes of the different errors have been analysed.

**Figure 6:**
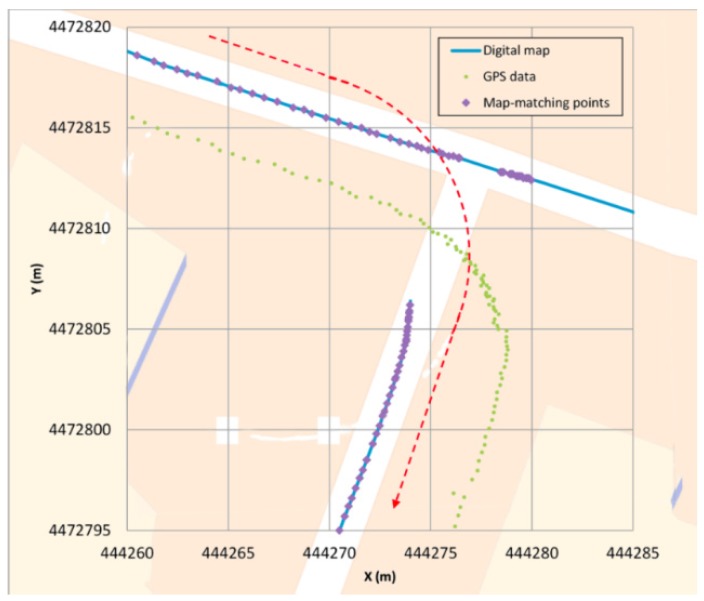
Type I error example.

**Figure 7:**
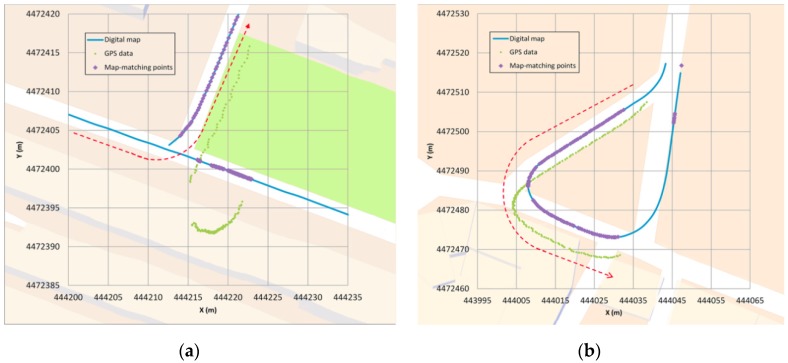
Type II error examples. (**a**) T-junction; (**b**) V-junction.

**Figure 8:**
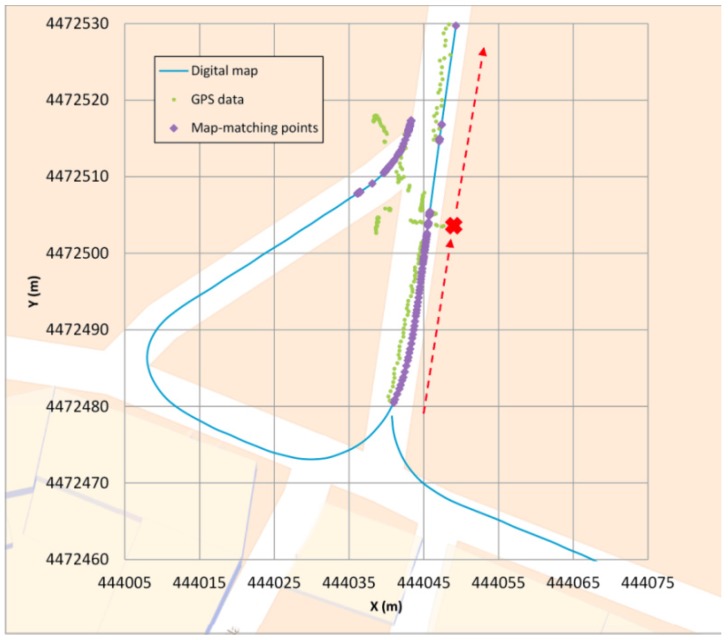
Type III error example.

Table 2 shows the results for tests on the two test areas and the kind of errors detected. The causes of the errors are quite specific and their frequency very low. Besides, the time needed to restore the correct positioning is quite short. The main errors that appear can be divided into three different types:

- Type I: The first error (Figure 6) occurs when a junction is crossed and a legal turn is made instead of going straight. Since the priority link at this scenario is, in general, the one that involves going straight (hence, getting a higher belonging value) if the positioning generated by the GPS is not quite accurate, it could occur that for a few GPS points the algorithm locates the vehicle in an incorrect link (the one that goes straight). However, when the direction of the GPS points is parallel to the segment where the vehicle actually is, and when the vehicle moves away from the junction, the algorithm changes and locates the vehicle in the correct link. This error is quite unusual and the algorithm restores the correct positioning in a short period of time.
- Type II: The second error (Figure 7) occurs when the GPS coverage is lost for a period of time and gets back near a junction and the algorithm decides incorrectly which segment is the good one after the vehicle turn. In this case, the map-matching process is performed as if it were the initial point. In some cases, this causes a mismatching, although after a few seconds the algorithm corrects its mistake locating the vehicle in the correct link. After losing the GPS signal, if the vehicle is in another segment when the signal is recovered and the belonging value does not decrease its weight, this could make the algorithm mismatch the position of the vehicle. After GPS positioning is recovered, the algorithm locates the vehicle in the incorrect segment because it is a priority link (taking into account the segment where the vehicle was located before) and after a few points it changes to the other segment, which is obviously where the car is. This error is quite unusual and the algorithm restores the correct positioning in a short period of time.
- Type III: The third error (Figure 8) occurs when the vehicle is not moving. In these cases the GPS position fixes could appear scattered and if the vehicle is near a junction, they could get closer to another link than the one in which the vehicle is stopped, eventually changing its matching position. Also, if the stop takes a long time, when the vehicle starts to move again the algorithm is not yet considering the possibility of relocating the vehicle (this only happens for a few seconds after a change of segment is made) so it will take more time to relocate the vehicle correctly than in the other errors. It is important to point out that this kind of error can be solved by taking into account the speed information, even if it is not too accurate. It should also be pointed out that the 11.8 s error that appears in Table 3 does not occur when the vehicle is moving but is solved when the vehicle starts moving, but it takes more time than in the other errors to relocate the vehicle (around 3 s).

**Table 3:** Map-matching algorithm results.

	Test Area 1	Test Area 2	
Test duration (s)	1060.0	1050.1	
GPS signal availability (%)	61.2%	61.1%	
--	# error appears (without speed information / with speed information)	# error appears (without speed information / with speed information)	longest duration (s)
Error type I	4 / 4	2 / 2	2.1 / 2.1
Error type II	3 / 3	2 / 2	1.6 / 1.6
Error type III	7 / 2	3 / 2	11.8 / 3.0

To conclude, some characteristics regarding the performance of the algorithm could be pointed out:

- The algorithm does not mismatch the vehicle’s position when the GPS inputs have large errors in positioning.
- After a change of segment when locating the vehicle, the algorithm considers the possibility of being mistaken and analyses other solutions for a few points after the change is made. Therefore, this allows it to relocate the vehicle if necessary.
- Knowing that the vehicle is stopped could significantly improve the results, even without knowing the speed of the vehicle with high accuracy. This allows the algorithm to eliminate the error that can be introduced by the spreading of the GPS signal, which in bad conditions of GPS coverage could be quite high.
- The perpendicular distance value penalizes those digital maps with a high density of points, that is, with little distance between them, as in the map used in this research, improving the results if this distance is higher, which is more usual. However, the algorithm proved to be robust even in these unpromising circumstances.

## 4. Conclusions

A map-matching algorithm that does not require inertial sensors and performs well even in areas with low GNSS coverage is put forward in this research. It has been observed that the errors that appear have a repetitive pattern and that these errors are corrected in a few seconds, which means the user does not notice them. Also, it can solve a common problem that navigator systems have, in which once the system has decided to locate the vehicle in a link, it takes time for it to relocate the vehicle in the event of a mismatch. After a change of segment, this new algorithm considers the possible alternatives that existed before the change, relocating the vehicle if necessary, when the results of the weights seem more coherent for the vehicle to be located in another link. This makes the correction process quicker than with other algorithms.

The algorithm presents limitations in complex scenarios with Y-junctions and frequent signal reception losses that sometimes make it mismatch the vehicle position. Nevertheless, the results obtained are very satisfactory and the corrections, in the event of there being an error, are carried out quickly, thus, avoiding the need to use inertial sensors.

The map-matching algorithm is also valid for commercial digital maps. Considering the analysis of the most frequent errors detected, their main cause is the degradation of the GNSS signal while the digital map accuracy is responsible only for a reduced set of incorrect locations. Obviously, in the event that the map has worse accuracy, vehicle positioning could be negatively influenced but the structure of the algorithm could provide better results than other methods that work in real time.

Finally, it should be pointed out that in spite of being appropriate for informative functions, these algorithms are not useful for applications where safety is a critical element. In those kinds of applications more accuracy is needed, both in the digital map data and in positioning the vehicles into them. The map-matching algorithms, as conceived today, will not make sense in these scenarios because there will be more accurate maps and also more accurate satellite positioning, making locating the vehicle easier for the algorithm. Nonetheless, to achieve these values of accuracy in the automotive field is a different technological problem. Therefore, as of today, it is necessary to turn to these map-matching algorithms that try to absorb and minimize the limitations of positioning systems and digital maps.
